# Polymer–Lipid Pharmaceutical Nanocarriers: Innovations by New Formulations and Production Technologies

**DOI:** 10.3390/pharmaceutics13020198

**Published:** 2021-02-02

**Authors:** Sabrina Bochicchio, Gaetano Lamberti, Anna Angela Barba

**Affiliations:** 1 Eng4Life Srl, Spin-Off Accademico, Via Fiorentino, 32, 83100 Avellino, Italy; sbochicchio@eng4life.it (S.B.); glamberti@unisa.it (G.L.); 2 Dipartimento di Ingegneria Industriale, Università Degli Studi di Salerno, Via Giovanni Paolo II, 132, 84084 Fisciano, Italy; 3 Dipartimento di Farmacia, Università Degli Studi di Salerno, Via Giovanni Paolo II, 132, 84084 Fisciano, Italy

**Keywords:** polymers, lipids, hybrid nanoparticles, nanotechnologies, production technologies, drug delivery

## Abstract

Some issues in pharmaceutical therapies such as instability, poor membrane permeability, and bioavailability of drugs can be solved by the design of suitable delivery systems based on the combination of two pillar classes of ingredients: polymers and lipids. At the same time, modern technologies are required to overcome production limitations (low productivity, high energy consumption, expensive setup, long process times) to pass at the industrial level. In this paper, a summary of applications of polymeric and lipid materials combined as nanostructures (hybrid nanocarriers) is reported. Then, recent techniques adopted in the production of hybrid nanoparticles are discussed, highlighting limitations still present that hold back the industrial implementation.

## 1. Introduction

Nanoparticle (NP) technology represents a revolutionary drug delivery platform that enhances the conveyance of active molecules to maximize their therapeutic index and to minimize undesirable side-effects, improving the treatment of several diseases [1,2]. In a scenario characterized by the rapid pharmaceutical development of new drug products such as gene silencing molecules based on RNA interference, recombinant proteins/peptides, and other promising biotech-therapeutics, NP engineering stands as an advanced strategy that, projected towards the continuous optimization of carrier material; size; and physical, chemical and structural properties, allows us to overcome issues of instability, biocompatibility, poor membrane permeability, and bioavailability of drugs, improving their pharmacokinetics and pharmacodynamics.

The biomimetic behavior of the lipid materials and the mechanical advantages linked to the polymeric materials are the strengths of the latest generation nanocarrier systems. The latter are characterized by the combination of several kinds of natural/synthetic lipids and polymers [3], showing different properties (i.e., architectures, size, external charge, response to external stimuli) and being suitable for encapsulating a multitude of active molecules that cannot be delivered effectively through conventional routes and could not even be efficiently encapsulated in a carrier system entirely composed of lipid or polymeric materials. Biocompatibility is a topic of spirited discussion and relevance.

**Figure 1:**
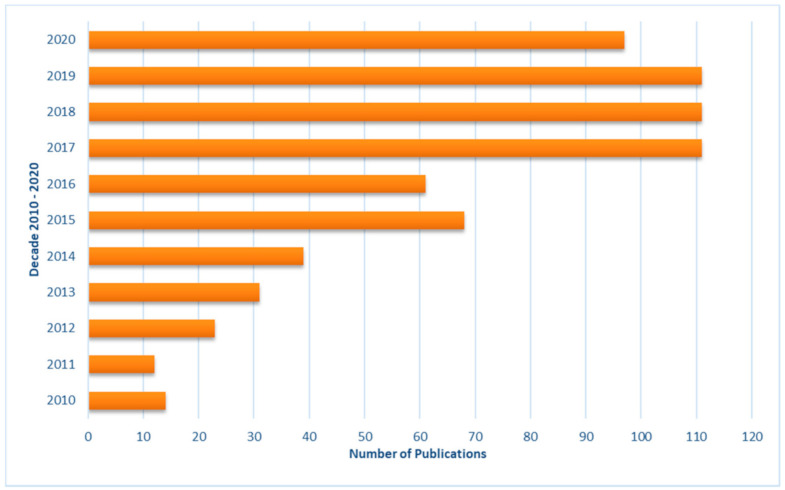
Number of publications from the years 2010 to 2020 (source: Web of Science Core Collection, query search: “lipid polymer hybrid nanoparticles”, last accessed 27 December 2020).

Indeed, each with its own peculiarities, these lipid polymeric nanoparticles (LPNs), including polymer-modified liposomes [4,5], polymeric micelles [6], surface-modified solid lipid nanoparticles (SMSLNs) [7] lipid–polymer hybrid nanoparticles (LPHNs) [8], and other combinations of polymer and lipid building blocks, allow for the overcoming of the non-negligible limitations of “pure nanoparticles” totally composed of lipids (i.e., liposomes) or polymers, offering countless advantages in various fields of applications such as biomedical, pharmaceutical, and nutraceutical spheres. The interest of the scientific community in these hybrid systems has increased tremendously over the last decade, as attested by the number of papers published in last 10 years, with more than 670 products focused on the concept of lipid polymer hybrid nanostructures (see Figure 1).

Under formulation point of view, one key point about the use of lipids and polymers to design and to realize nano-delivery systems is the biocompatibility of the starting ingredients. Biocompatibility is defined as the “*ability of a biomaterial to perform its desired functions … without eliciting any undesirable effect* …” [9]. The biocompatibility should be tested by in vivo experiments, which, however, are costly and have ethical issues. Therefore, several in vitro methods have been defined and used, mainly cytotoxicity and MTT (3-(4,5-dimethylthiazol-2-yl)-2,5-diphenyltetrazolium bromide) assays, to assess the cell metabolic activity [9]. For commonly used polymers and lipids, the biocompatibility is well studied and well known (readers can refer to recent reviews, for polymers [10], as well as for lipids [11]). Lipids (and in particular the membrane-forming phospholipids) are inherently well tolerated, since they are the natural constituent of living cell membranes. Among them, the cationic phospholipids, which are the ideal candidates to build delivery systems for negatively charged molecules (nucleic acid-based drugs, for example, such as mRNA and short interfering RNA (siRNA)), may pose biocompatibility issues. Therefore, for cationic lipids and cationic liposomes, the biocompatibility is often the starting point for the research, even before to test their usability [12,13].

Under the production point of view, the advancements in design of performant LPN blended delivery systems require a continuous improvement of the production strategies that can be easily adopted at an industrial level to effectively translate the potential of these smart particles from the laboratory to the market. To date, the greatest limitation in the production of these sophisticated systems lies precisely in the use of conventional techniques that do not reflect industrial needs. Indeed, traditional batch techniques, operating on small volumes and requiring long preparation times, without offering a control over the final carriers features, must be urgently replaced by versatile and economic methodologies, which, working in continuous, can allow for rapid and massive production.

This work is focused on products and production technologies, at the nanoscale, involving lipid and polymeric materials. It is structured in two sections. In the first section, features of representative polymeric and lipid nanoparticles are briefly introduced. Then, examples of blended nanometric carriers obtained by lipid–polymeric technologies are presented in terms of structural and functional main features. In the second section, the problem related to the use of “ancient” techniques to produce the increasingly sophisticated “new generation” delivery systems is emphasized by highlighting the advantages and disadvantages of the conventional and latest used methodologies.

## 2. Polymeric, Lipid, and Hybrid Nanostructures

Polymeric nanoparticles (PNs) are biodegradable delivery systems with several interesting properties that strictly depend on the type of matter they are made up of [14]. They can be composed of natural (i.e., chitosan, hyaluronic acid, starch, dextran, alginate, albumin, and heparin), semi-synthetic (i.e., poly(methacrylic acid) and polysorbate 80-grafted starch), or synthetic polymers (i.e., PLGA, PLA, PCL, PEG, PHEMA, PHPMA, PVA, PNIPAm, amphiphilic polymer, polystyrene, PAA, PEI, and PBAE—see Table 1 for the full names) and can assume different architectures such as solid nanoparticles, core–shell structures, polymeric micelles, and polyplexes [3,15,16,17,18]. The notable advantages of PNs lie in their great stability (higher than that possessed by lipid particles), which guarantees a sustained drug delivery, and in their stimuli-responsive properties (such as temperature, pH, redox, ionic strength), which allow for the obtaining of a precise control over the intracellular release of the active molecule [4,19]. However, due to the poor biocompatibility and the low affinity to the cell membrane, PNs present a low cell interaction, resulting in a poor drug delivery efficiency [5]. To overcome these specific lacks, polymeric nanoparticles have recently been used as a “bearing structure” for a revolutionary class of biomimetic delivery systems. In particular, we applied the idea to use cell membranes for coating nanoparticles in an attempt to mimic the ability of cells to interface and interact with physiological environments [20]. This approach falls inside the development of biomimetic nanotechnologies based on the “camouflaging” methods. The biomimetic NPs not only retain the physicochemical features of the synthetic vehicles but also inherit the cell membranes’ intrinsic functionalities [21,22]. As examples, erythrocytes (red blood cell (RBC) membrane-camouflaged nanocarriers (RBC-MCNs)) are used in studies for tunable paclitaxel (PTX) release kinetics from biocompatible isotactic and atactic polylactide (PLA) polymeric vesicles [23] and 5-fluorouracil (5-FU) delivery from by chitosan-coated poly(lactide-co-glycolic acid) nanoparticles [24].

Lipid nanoparticles (LNs) are safe and effective carriers made from natural or synthetic lipids, among which the ones generally most used are fatty acids (i.e., palmitic, myristic, and stearic acids), glycerides (i.e., glyceryl monostearate and caprate), steroids (i.e., cholesterol (Chol)), phospholipids (i.e., PC, DPPC, DSPE, DMPC, DOPE, DOPC, POPC, HSPC, PE, and lecithin—see Table 2 for the full names), and other lipids such as 1,2-dioleoyl-3-trimethylammonium propane (DOTAP) and 1,2-dipalmitoyl-3-trimethylammonium-propane (DPTAP) [3,18,25].

In particular, LNs can assume disparate structures and can be classified into solid lipid nanoparticles (SLNs), nanostructured lipid carriers (NCLs), lipid nanocapsules (LNCs), micelles, and liposomes [26]. LNs are highly appreciated for their extraordinary biocompatibility, higher than that of polymeric particles, which allows for a prompt cellular uptake of the therapeutic substances. However, lipid particles show relevant limitations given their poor drug loading capacity, which is often restricted by the solubility of drug in the lipid melt, the rapid drug loss due to the structure of the lipid matrix, its expulsion during storage, the tendency to aggregate, as well as a high instability in biological fluids [27,28]. An emblematic example is represented by liposomes that, although reflecting the main features of an ideal drug delivery system [29] and being in great demand for their large affinity for plasma membrane due to their remarkable biomimetic properties, are characterized by several issues related to the rapid degradation by the reticuloendothelial system (RES), the difficulty to achieve a sustained drug delivery for long periods of time, and the tendency to aggregate with serum proteins (typical of cationic liposomes) [20].

**Figure 2:**
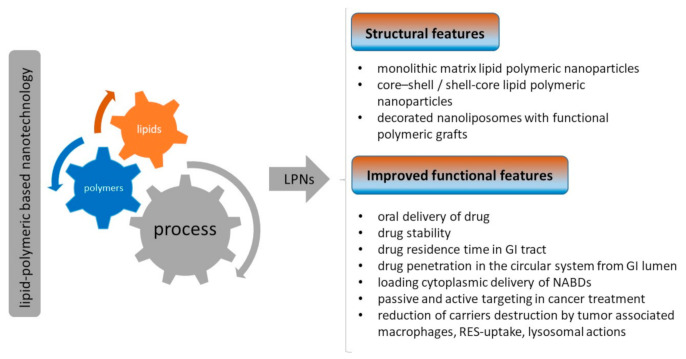
Schematic representation of structures and potential improvements of functional features of lipid–polymeric nanoparticles for pharmaceutical purposes.

In light of the above, in recent years, an attempt has been made to merge the advantages of polymeric and lipid materials in a single smart carrier system by suitable technique and through a careful selection of biocompatible polymer–lipid combinations, which guarantee a high affinity with biological membranes, allow a controlled drug release over a prolonged period of time, and provide the possibility to co-encapsulate therapeutics with different properties [30,31] (conceptualization schemed in Figure 2).

Among these new blended particles, the most explored and appreciated are the lipid–polymer hybrid nanoparticles (LPHNs) and the liposomes covered with polymers, especially chitosan, carriers capable of loading and transporting a wide range of functional molecules from anticancer to vitamins, peptides, proteins, gene material (see also in following paragraphs), metallic inclusions, cells, and other therapeutics [32] (in Table 3 are several examples of LPHNs loaded with different active ingredients and produced by different preparation methods).

**Figure 3:**
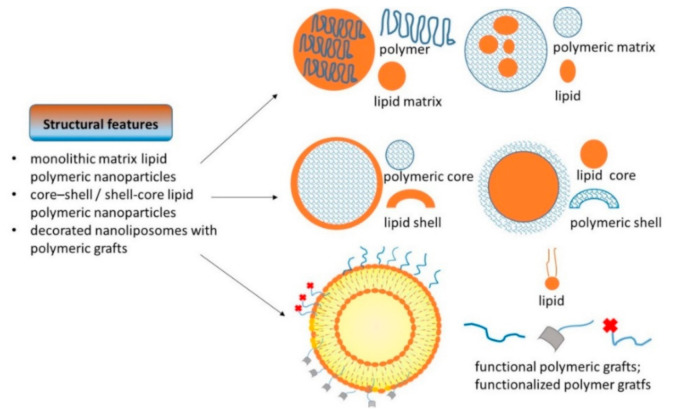
Simplified schematic representation of structural features of lipid–polymeric nanoparticles.

The lipid–polymeric-based technology can concern the modification of single lipids (i.e., polyethyleneimine (PEI) stearate, poly(lactic-co-glycolic acid) (PLGA) lipid, polyethylene glycol (PEG) lipid) [30] or of entire lipid/polymeric particles with the consequent production of hybrid systems that, according to their architecture and manufacturing process, can be classified into monolithic matrix lipid polymeric nanoparticles, in which a lipid phase contains an homogeneously dispersed polymeric drug complex (or otherwise a polymeric core covered by a lipid shell); core–shell lipid polymeric nanoparticles, characterized by a polymeric core surrounded by a lipid shell (or otherwise a lipid core covered by a polymer shell); and liposomes, whose surface is decorated with polymeric materials [2,3,30,32,33,34] (a simplified schematic representation in Figure 3).

Release properties and payload entity of LPHNs are based on features of the used ingredients and on architecture of the final delivery structure. Mechanisms of release such as erosion and diffusion, and actions such as membrane fusion, endocytosis, and passive and active targeting are also inducible and tunable by suitable formulations that can also confer the capability to respond to external physical stimuli (stress, electricity, light irradiation) or to internal chemical or biochemical triggers (pH, metabolites, antigens, or enzyme presences) [8,26,33,35].

LPHNs find their application in various fields. First, they have been applied in the pharmaceutical field, improving the oral delivery of drugs, increasing their stability; prolonging their residence time in the gastrointestinal (GI) tract; increasing their penetration in the circular system from GI lumen [30]; and improving the systemic delivery of several active molecules, i.e., increasing the loading and ameliorating the cytoplasmic delivery of nucleic acid-based drugs (NABDs) [3,36]. LPHNs play a crucial role in the field of cancer treatment for the delivery of cytotoxic drugs (i.e., hormonal therapy, gene targeted therapy, immunotherapy) by improving the passive and active targeting of the loaded drug, and overcoming the limitations related to the therapeutics off-target accumulation, carrier destruction by tumor-associated macrophages, RES uptake, and the lysosomal degradation of nanocarriers [28,37].

**Table 3:** Examples of lipid–polymeric hybrid nanoparticles loaded with different active ingredients and by different preparation methods.

LPHN Composition/*Active Ingredient*/Preparative Method	Application	Reference
Lipid, lipoid S75-chitosan, low molecular weight (monolithic nanostructures)/*cisplatin*/ionic gelation (ethanolic solution dropped in chitosan acidulate solution with cisplatin)	cancer therapies	Khan et al., 2019 [38]
Nanoparticles with PLGA core layered by lecithin lipid PEG modified with iRGD peptides/*isoliquiritingenin*/modified single-step nanoprecipitation	breast tumor therapy	Gao et al., 2017 [39]
Poly(lactide-*co*-glycolic acid) layered by chitosan, erythrocyte membrane*/*fluorouracil*/double emulsion (w/o/w) method (5-FU aqueous solution in PLGA-dichloromethane and, finally, dropped into an aqueous phase) *NEs were added by incubation into 5-FU polymeric nanoparticle suspension 1,2-Dipalmitoyl-snglycero-3-phosphocholine, cholesterol, erythrocyte membrane*/*fluorouracil*/thin film hydration method—liposomes; liposomes incubated in chitosan acidulate solution then membrane extrusion methods—chitosomes, i.e., liposomes with chitosan coating *NEs were added by incubation into 5-FU nanochitosome suspension	liver cancer therapies	AlQahtani et al., 2021 [24]
Ionizable lipid L319, distearoylphosphatidylcholine, cholesterol, and 1,2-dimyristoyl-rac-glycero-3-methoxy-PEG (nanoliposomes PEGylated)/*siRNA*/spontaneous vesicle formations method by pumping ethanolic solution with lipids and citrate buffer solution with siRNA within a chromatography tubing	vaccine therapy against SARS-CoV-2	Polack et al., 2020 [40] Walsh et al., 2020 [41] Maier et al., 2013 [42] Pardi et al., 2015 [43]
1,2-Distearoyl-sn-glycero-3-phosphoethanolamine—PEG, gelucire, PLA, (fructose-tethered phospholipid coated lipophilic polymeric core)/*beta carotene and methotrexate*/modified single-step nanoprecipitation method	breast cancer therapy	Jain et al. 2017 [44]
L-α-Phosphatidylcholine, cholesterol, chitosan (nanoliposomes coated by chitosan)/*indomethacin*/simil-microfluidic method	cancer therapies	Dalmoro et al., 2018 [45]
L-α-Phosphatidylcholine, cholesterol, chitosan (nanoliposomes coated by chitosan)/*D3, K2 vitamins*/simil-microfluidic method	food supplements	Dalmoro et al., 2019 [46]
PLA, soya lecithin, stearylamine (lipid shell, polymer core with antimicrobic)/*norfloxacin*/emulsification solvent evaporation method	antimicrobial therapies	Dave et al., 2017 [47]
PLGA, soybean lecithin, and PEG (polymer core-encapsulating gold crystals, lipid monolayer surrounding, outer lipid PEG)/g*old nanocrystals*/nanopreciptitation method	bioimaging purposes	Mukherjee et al., 2019 [48]

### Examples of Application

Recent literature providing promising approaches are discussed in the following section. In Table 3, examples of lipid–polymeric hybrid nanoparticles (composition and field of application) are shortly summarized.

In 2019, Khan and collaborators produced and tested cisplatin-loaded lipid–chitosan hybrid nanoparticles, demonstrating a supported controlled delivery of the drug with enhanced mean residence time and half-life in rabbits, suggesting the promising application of these new LPHNs for the controlled delivery of cisplatin in cancer therapy [38].

In 2021, AlQahtani and collaborators, in order to contrast liver cancer, developed a membrane using hybrid NPs coated with nanoerythrocytes (NEs) with the aim of prolonging the 5-FU delivery circulation time. Several formulations of hybrid biomimetic nanocarriers have been used for in vitro and in vivo tests: NE-decorated, 5-FU-loaded, chitosan-coated poly(lactide-co-glycolic acid) nanoparticles (5-FU-C-NPs-NEs); chitosomes—chitosan in combination with liposomes made by 1,2-dipalmitoyl-sn-glycero-3-phosphocholine (DPPC) and cholesterol as lipids—(5-FU-C-LPsNEs); and 5-FU-NEs (nanostructures without NEs were also prepared as control). The achieved results revealed that 5-FU-C-NPs-NEs showed the most desirable characteristics in terms of sustained release profile, drug load efficiency, and retention of erythrocyte membrane properties [24].

In the work of Gao and collaborators (2017), in order to improve the therapeutic outcome of isoliquiritigenin, the authors developed a natural anti-breast cancer dietary compound characterized by low bioavailability, a tumor-targeting lipid–polymer hybrid nanoparticle system composed of a polymeric PLGA core coated with a layer of lecithin lipids, and an additional PEG film modified by the iRGD (9-amino acid cyclic) peptides. The in vitro and in vivo studies’ outcome showed that the developed nanoparticles did not have damaging effects on normal tissue, and increased the cell-uptake efficiency and improved the targeting ability with an accumulation in breast tumors [39].

It is well known that the recent vaccine therapy against SARS-COV-2 (severe acute respiratory syndrome-coronavirus-2), as reported in Polak and collaborators (2020) and Walsh and collaborators (2020), is based on mRNA molecule delivered by LNs [40,41]. The vaccine, commercially known as Comirnaty, is based on a LN technology that has been pointed out in recent years [42,43]. It is based on a combination of four components: an ionizable lipid designed for the purpose of increasing bioavailability by a tailored biodegradation (cleavage), a PEGylated molecule to modulate the half-life of the formulation, and the common components of liposomes (phosphatidylcholine and cholesterol). The approach is the most promising for the delivery of drugs that are based on RNA molecules, particularly for vaccines [49], which are, currently, a highly topical issue.

The targeting potential of new fructose-tethered lipid-polymeric hybrid nanoparticles (F-BC-MTX-LPHNPs) co-loaded with beta carotene (BC) and methotrexate (MTX), for intravenous administration, was also investigated by Jain et al. (2017), showing the ability of these systems to selectively convey the chemotherapeutic agent to the breast cancers, with an improvement of MTX-induced hepatic and renal toxicity due to the co-delivery of BC [44]. Finally, in a recent work of Dalmoro and collaborators (2018) [45], chitosan–lipid hybrid delivery systems for indomethacin dosage, to be used for the oral-controlled release of the drug, were produced, showing a gastro-retentive behavior in simulated gastric and intestinal fluids.

It should be noted that, in addition to the oral and systemic traditional pathways, LPNs can be administered by alternative routes such as ocular, pulmonary, rectal, nasal, buccal, sublingual, and transdermal routes, expanding their fields of application and patient compliance [50,51,52]. By way of example, buccal adhesive drug delivery systems, exploiting polymers as the adhesive component (i.e., chitosan and polyacrylates), have been studied for the production of water-soluble bioadhesive formulations, with a strong affinity for mucosal membranes [53]. In that regard, in a work of Dalmoro and coworkers (2019), polymer–lipid hybrid nanoparticles, encapsulating vitamin D3 and vitamin K2, with improved features in terms of stability, loading, and mucoadhesiveness, were produced for potential nutraceutical and pharmaceutical applications. The work, which concerns the production of liposomes coated with chitosan, is deepened in the next section in terms of the innovativeness of the production technique presented [46].

In the dermatological field, LPNs are highly appreciated for the treatment of several topical infections caused by bacteria, fungi, surgery, and accidental injury or abrasion [54]. In a work of Dave and coworkers (2017), PLA and soya lecithin-based lipid–polymer hybrid nanoparticles were prepared for the topical and site targeting delivery of norfloxacin and tested for their antimicrobial properties against *Staphylococcus aureus* and *Pseudomonas aeruginosa,* showing their high potential for usage as a topical antibiotic drug carriers [47].

Moreover, with the growing interest in health/enriched/functional foods, polymer–lipid hybrid systems are also highly desirable in nutraceuticals and food supplement fields to exceed the restrictions of poor water solubility, low bioavailability, bad taste and aroma, pH sensitivity, and easy degradation of a wide number of active molecules [55]. Finally, LPNs also find application in the bioimaging field as delivery systems for contrast agents (diagnostic tools) [48,56].

## 3. Innovations and Performances of Production Technologies

In actuality, the setup of more effective scaled-up industrial processes, by exploring new and sustainable technologies or re-adapting conventional ones to the chemistry of new materials, is one of the main objectives of the scientific community, aimed at translating the innovations from the laboratory to the market. Indeed, one of the major problems and controversies related to the field of nanotechnologies applied to drug delivery lies within the limits of the “old” conventional techniques currently used for the production of smart and “innovative” systems.

Conventional techniques such as nanoprecipitation [48], emulsification-solvent evaporation [8], mechanical mixing, and dropwise [57,58] (i.e., the layer-by layer (LbL) [59,60]) constitute, to date, the most used techniques for the preparation of lipid–polymeric nanocomplexes, including LPHNPs and polymer-coated liposomes (brief notes on techniques are summarized in Table 4). LPHNPs, characterized by PEG surface surrounding a lipid layer that contains a polymer core encapsulating the drug, are generally prepared by the mechanical mixing of preformed polymeric particles with a dried lipid film or, directly, with lipid particles previously produced by maintaining the temperature above that of lipids phase transition. Subsequently, in order to obtain hybrid nanoparticles of homogeneous dimensions, researchers must adopt homogenization or extrusion processes [48].

The nanoprecipitation and the emulsification-solvent evaporation methods are the most used techniques for the production of LPHNPs. Briefly, nanoprecipitation consists in the dropwise addition of the polymer, previously dispersed in a water-miscible organic solvent containing the drug, to an aqueous dispersion of lipid under continuous stirring. LPHNPs are formed after the organic solvent evaporation [61]. Examples of the recent application of this technique, with some modifications, are found in the work of Dehaini et al. (2016), wherein ultra-small lipid–polymer hybrid nanoparticles for the tumor-penetrating delivery of docetaxel were produced [62]. Liu and coworkers used this method for the production of paclitaxel- and triptolide-coloaded lipid–polymer hybrid nanoparticles for lung cancer therapy [63], while Tahir et al. produced methotrexate-loaded LPHNPs through a single-step modified nanoprecipitation method [64].

In the emulsification-solvent evaporation, LPHNPs are formed through an oil-in-water (o/w) emulsion or through a w/o/w (water/oil/water) emulsion, as detailed in [48]. By this method, Dave and collaborators (2017) recently produced lipid–polymer hybrid nanoparticles for the topical and site-targeting delivery of norfloxacin to be used as topical drug delivery systems [47]. Leng et al. (2018) used this method for the fabrication of budesonide-loaded LPHNPs, on the basis of PLGA and the cationic lipid DOTAP, which could be combined with siRNA for the cure of chronic obstructive pulmonary disease [65].

Despite the excellent results obtained in vitro and in vivo, which demonstrate the extraordinary performance of these carriers, the production methods do not allow a scale-up of the formulations whose advantages do not materialize on the market.

New emerging technologies are being explored for the production of LPHNPs and concern the spray drying [66,67], microfluidics [56,68,69], and its combination with multi-inlet vortex mixer (MIVM) systems (brief notes on techniques are summarized in Table 4). In 2019, Dormenval and collaborators used the spray drying method for the production of siRNA-loaded lipidoid–polymer hybrid nanoparticles for inhalation [70], and, in the same year, the herringbone-patterned 3D printed reactor was explored by Bokare and collaborators for the production of rifampicin-loaded LPHNPs [71].

Unfortunately, even these technologies are not very accessible given the high costs of the equipment used.

The picture is not very different in reference to polymer-coated liposomes, for which a promising novel technology has recently been developed, as described below. In the last decade, in the light of the medical goals achieved by pegylated liposomal-based therapeutics approved for clinical use, among which Onpattro (the first PEG lipid-based nanoformulation encapsulating siRNA that was approved in 2018 for the treatment of transthyretin amyloidosis) represents just one example [32,72,73], the scientific community has focused on the engineering of these promising lipid vesicles by combining them with polymeric materials to improve their performance. Given the huge potentiality of polymer-modified lipid vesicles in different fields of application, there is a strong interest in increasing their production.

To date, liposome polymeric coating is carried out through conventional methods such as the simple incubation of a liposomal suspension with a polymeric solution [74,75] or through the dropwise method, which in the early 1990s was explored for the coating of liposomes with chitosan [76], is still the most popular form for the production of these carrier systems. The strategy involves the dropwise addition of a polymeric solution, through the use of a syringe pump, to a preformed liposomal suspension, while stirring [77], or, on the contrary, the polymeric solution is added drop by drop to a previously produced liposome solution [78].

Over the years, the technique has led to the production of coated liposomes, mainly of the chitosan polymer, for the delivery of various active molecules such as indomethacin [79], clotrimazole [80], resveratrol [81], doxorubicin [56,82] quercetin [83], proteins [84,85], curcumin [78], and other therapeutics [86,87], but the fabrication processes have remained at the bench-scale, leading to the production of restricted volumes in output. In addition to the low productivity, being a bulk technique, it is also characterized by a poor control over the final features of the covered lipid vesicles and requires high energy and long periods of time for process.

An innovative technique focused on supercritical reverse-phase evaporation technology was explored in 2006 for the production of chitosan-coated cationic liposomes, but involved an expensive apparatus that does not meet the industrial needs [88].

Recently, in order to overcome the aforementioned problems and with a focus on a cheap and easy plant setup, the authors of [77] presented a novel liposome-covering method based on microfluidic principles, with great potential for the industrial sector. In particular, a semi-continuous setup, whose layout is described in [89], was firstly developed for the production of lipid vesicles and then adapted for the fabrication of chitosan-covered liposomes, as detailed in [77]. Briefly, the coating process consists of the realization of a contact between two flows, a previously prepared nanoliposomes suspension and a chitosan solution, which are pushed through peristaltic pumps (at equal volumetric flow rates) into the production section where their continuous and uniform interaction leads to the production of completely covered liposomal systems, characterized by a core–shell structure. In Reference [59], in a comparison between this novel method and the conventional dropwise technique in terms of phenomenological aspects underlying the formation of chitosan-covered nanoliposomes, the product quality and the manufacturing yield obtained is presented, underlining the innovativeness of the simil-microfluidic process in reducing the cost impact on manufacturing and guaranteeing a massive production yield [77]. This novel method has been applied for covering nanoliposomes containing indomethacin as the active ingredient, obtaining nanoliposomes characterized by high loading and drug encapsulation efficiency, with a chitosan layer thicker and smoother than that achievable by the dropwise method [45]. Moreover, by the same technology, stable and mucoadhesive chitosan-coated liposomal carriers, loaded with vitamin D3 and K2, were produced for nutraceutical purpose [46]. Finally, in 2019, the novel simil-microfluidic technique was used to produce a vegan alternative formulation composed of cholesterol-free liposomes coated by guar hydroxypropyltrimonium chloride (Guar-HC) obtaining stable mucoadhesive carrier systems without aggregation phenomena [90].

**Table 4:** Several examples of lipid–polymeric hybrid nanoparticle production techniques (schematizations and procedures are shown in the reported references).

LPHN Production Techniques/*Output Structure*	Main Advantages	Main Disadvantages	References
*Conventional techniques*			
Nanoprecipitation method/*monolithic nanosystems*	easy to perform, inexpensive	bulk technique (bench-scale), long process times, limited output volumes, poor control	Mukherejee et al., 2019 [48] Dehaini et al., 2016 [62] Zhang et al., 2010 [8]
Dropwise (layer-by-layer) method/*coated nanosystems*	easy to perform, inexpensive	bulk technique (bench-scale), long process times, limited output volumes, poor control	Hasan et al., 2016 [57] Tan et al., 2016 [58] Bochicchio et al., 2018 [77]
Emulsification-solvent evaporation method/*coated nanosystems*	easy to perform, inexpensive	bulk technique (bench-scale), long process times, limited output volumes, poor control	Mukherejee et al., 2019 [48] Zhang et al., 2010
*Emerging technologies*			
Spray drying/*monolithic or layered nanosystems*	continuous technique, massive production, precise control	high energy consumption	Dormenval et al., 2019 [70]
Microfluidic method/*layered* *polymeric core, outer lipid–PEG*	precise control	limited output volumes (bench-scale), expensive apparatus	Wei et al., 2020 [69] Mieszawska et al., 2013 [56]
Simil-microfluidic method/*chitosan or guar gum-coated nanoliposomes*	continuous technique, massive production, precise control, operative room conditions	presence of residual solvent	Bochicchio et al., 2018 [77] Barba et al., 2019 [90]
Super critical fluid technique/*chitosan-coated liposomes*	organic solvent-free preparations	expensive apparatus, high energy consumption	Otake et al., 2006 [88]

## 4. Conclusions and Perspectives

In this article, a concise overview of the potential of lipid–polymer hybrid nanocarriers was presented. Examples of improved characteristics in terms of functional activity of polymer–lipid nanostructures discussed in the literature were reported. The recent techniques adopted in the production of hybrid nanoparticles were briefly described, underlining the shortcomings still present (limited output volumes, low productivity being bulk techniques, high energy consumption, expensive configuration, long process times) to make the translation of the production of these intelligent particles from the laboratory to the market be sustainable, easy, and fast.

The current scenario on the topic of hybrid nanoparticles reveals a powerful potential of these conceptualized vectors—their uses can range from cancer treatments to nutraceutical, cosmeceutical purposes and diagnostic applications.

From a formulation point of view, research is conducting a careful selection of biocompatible polymer–lipid combinations through more extensive in vivo experiments (about 100 articles have been published on this topic in the last decade—source: Web of Science, Core Collection 2000–2021 query search: “biocompatible polymer–lipid AND in vivo”, last access 19 January 2021).

From a general production point of view, specific impasses in the production of LPHNs are linked to their stability and manipulation. Liquid suspensions are the most diffuse form of the hybrid nanoparticle preparations; otherwise a solid state can be achieved but often after further preparative steps such as, for example, lyophilization. Liquid suspension status often can constitute a limitation for stability and manipulation issues (undesired aggregation and microbiological contamination effects, storage at low or very low temperature, loss of ingredient effectiveness by oxidation phenomena, etc.). To this aim, processes such as concentration (by solvent evaporation unit operation), liquid–solid separation (by mechanical operations), and drying (freeze drying, spray drying—the latter of which is also a main preparative process, as discussed in this work) are currently under investigation. Of course, the features of these post-treatments also must fall in the needs to have sustainable (technologically and in terms of resources) processes for the pharmaceutical industry modernization.

## Figures and Tables

**Table 1:** Full name of polymers cited in the text.

Abbreviation	Full Name
PLGA	poly(lactic-*co*-glycolic acid)
PLA	poly(lactic acid)
PCL	polycaprolactone
PEG	polyethylene glycol
PHEMA	poly hydroxyethyl methacrylate
PHPMA	poly(2-hydroxypropyl methacrylate)
PVA	polyvinyl alcohol
PNIPAm	poly(*N*-isopropylacrylamide)
PAA	poly(amidoamine)
PEI	polyethyleneimine
PBAE	poly-(β-amino ester)

**Table 2:** Full name of lipids cited in the text.

Abbreviation	Full Name
Chol	cholesterol
PC	soybean phosphatidylcholine
DPPC	1,2-dipalmitoyl-sn-glycero-3-phosphocholine
DSPE	1,2-distearoyl-sn-glycero-3-phosphoethanolamine
DSPC	1,2-distearoyl-sn-glycero-3-phosphocholine
DMPC	1,2-dimyristoleoyl-sn-glycero-3-ethylphosphocholine
DOPE	1,2-dioleoyl-sn-glycero-3-phosphoethanolamine
DOPC	1,2-dioleoyl-sn-glycero-3-phosphocholine
POPC	1-palmitoyl-2-oleoyl-sn-glycero-3-phosphocholine
HSPC	hydrogenated soy phosphatidylcholine
PE	phosphor-ethanolamine
DOTAP	1,2-dioleoyl-3-trimethylammonium propane
DPTAP	1,2-dipalmitoyl-3-trimethylammonium-propane

## Data Availability

Not applicable.

## Abbreviations

GI	gastrointestinal (tract, lumen)
LNCs	lipid nanocapsules
LNs	lipid nanoparticles
LPHNs	lipid–polymeric hybrid nanoparticles
LPNs	Lipid–polymeric nanoparticles
NABDs	nucleic acid-based drugs
NCLs	nanostructured lipid carriers
NEs	nanoerithrocytes
NP	nanoparticle
MTT	3-(4,5-dimethylthiazol-2-yl)-2,5-diphenyltetrazolium bromide
PNs	lipid nanoparticles
RBCs	red blood cells
RBC-MCNs	rbc-membrane-camouflaged nanocarriers
RES	reticuloendothelial system
siRNA	short interfering RNA
SLNs	solid lipid nanoparticles
SMSLNs	surface-modified solid lipid nanoparticles
